# Adaptive granularity in tensors: A quest for interpretable structure

**DOI:** 10.3389/fdata.2022.929511

**Published:** 2022-11-23

**Authors:** Ravdeep S. Pasricha, Ekta Gujral, Evangelos E. Papalexakis

**Affiliations:** Department of Computer Science and Engineering, University of California, Riverside, Riverside, CA, United States

**Keywords:** tensor, unsupervised learning, temporal granularity, tensor decomposition, multi-aspect data

## Abstract

Data collected at very frequent intervals is usually extremely sparse and has no structure that is exploitable by modern tensor decomposition algorithms. Thus, the utility of such tensors is low, in terms of the amount of interpretable and exploitable structure that one can extract from them. In this paper, we introduce the problem of finding a tensor of adaptive aggregated granularity that can be decomposed to reveal meaningful latent concepts (structures) from datasets that, in their original form, are not amenable to tensor analysis. Such datasets fall under the broad category of sparse point processes that evolve over space and/or time. To the best of our knowledge, this is the first work that explores adaptive granularity aggregation in tensors. Furthermore, we formally define the problem and discuss different definitions of “good structure” that are in practice and show that the optimal solution is of prohibitive combinatorial complexity. Subsequently, we propose an efficient and effective greedy algorithm called ICEBREAKER, which follows a number of intuitive decision criteria that locally maximize the “goodness of structure,” resulting in high-quality tensors. We evaluate our method on synthetic, semi-synthetic, and real datasets. In all the cases, our proposed method constructs tensors that have a very high structure quality.

## 1. Introduction

In the age of big data, applications deal with data collected at very fine-grained time intervals. In many real-world applications, the data collected spans a long duration and can be extremely sparse. For instance, a time-evolving social network that records interactions of users every second results in a very sparse adjacency matrix per second if observed at that granularity. Similarly, in spatio-temporal data, if one considers GPS data over time, discretizing GPS coordinates based on the observed granularity can lead to very sparse data which may not contain any visible and useful structure. How can we find meaningful and actionable structures in these types of data? Plenty of such datasets are multi-aspect in nature and hence can be modeled using tensors. For instance, a three-mode tensor can represent a time-evolving graph capturing user-user interactions over a period of time, measuring crime incidents in a city community area over a period of time (Smith et al., 2017), or measuring traffic patterns (Zheng et al., 2014). Tensor decomposition has been used in order to extract hidden patterns from such multi-aspect data (Kolda and Bader, 2009; Papalexakis et al., 2016; Sidiropoulos et al., 2017). However, the degree of sparsity in the tensor, which is a function of the granularity in which the tensor is formed, significantly affects the ability of the decomposition to discover a “meaningful” structure in the data.

**Figure 1:**
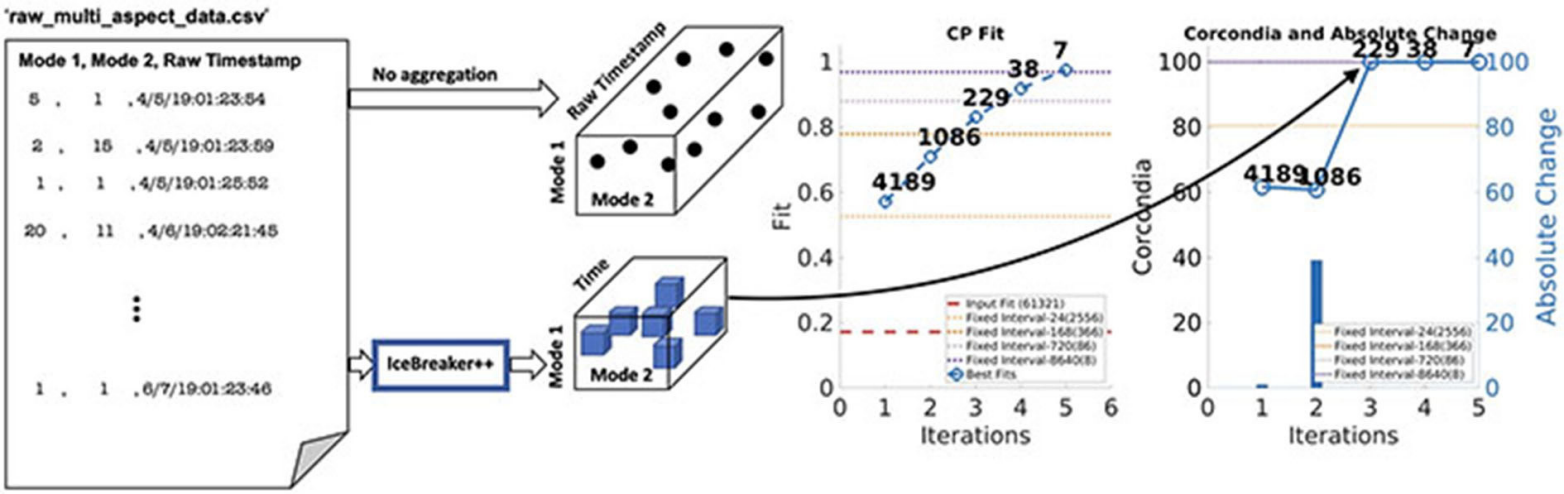
Starting from raw CSV files, ICEBREAKER++ discovers a tensor that has a good structure (under various measures of quality, including interpretability and predictive quality), outperforming traditional fixed aggregation heuristics. Furthermore, ICEBREAKER++ using various notions of locally optimal structure discovers different resolutions in the data.

Consider a dataset that can be modeled as a three-mode tensor, where the third mode is temporal as shown in Figure 1. If the granularity of the temporal mode is too fine (in milliseconds or seconds), one might end up with a tensor that is extremely long on the time mode and where each instance of time has a very small number of entries. This results in an extremely sparse tensor, which typically is of very high rank, and usually has no underlying exploitable structure for widely popular and successful tensor decomposition algorithms (Kolda and Bader, 2009; Papalexakis et al., 2016; Sidiropoulos et al., 2017). However, as we aggregate data points over time, the exploitable structure starts to appear (where-by “exploitable” means the kind of low-rank structure that a tensor decomposition can successfully model and extract). In this paper, we set out to identify what is the best such data-driven aggregation of a tensor which leads to better, exploitable, and interpretable structure, and how this fares against the traditional alternative of selecting a fixed interval for aggregation.

As far as tackling the problem above, there is a considerable amount of work that focuses on a special case, that of aggregating edges of a time evolving graph into “mature” adjacency matrices based on certain graph properties (Sun et al., 2007; Sulo et al., 2010; Soundarajan et al., 2016). In our work, however, we address the problem in more general terms, where the underlying data can be any point process that is observed over time and/or space, and where the aggregation/discretization of the corresponding dimensions directly affects our ability to extract interpretable patterns *via* tensor decomposition. Effectively, as shown in Figure 1, we work toward automating the data aggregation starting from raw data into a well-structured tensor. This paper is based on the preliminary work which has appeared in arxiv (earlier version arXiv:1912.09009v1; Pasricha et al., 2019) and non-archival workshop (Pasricha et al., 2020).

Our contributions to this work are as follows:

- **Novel problem formulation**: We formally define the problem of optimally aggregating a tensor, which is formed from raw sparse data in their original level of aggregation, into a tensor with exploitable and interpretable structure. We further show that solving this problem optimally is computationally intractable. To the best of our knowledge, this paper is the first to tackle this problem in its general form, and we view our formulation as the first step toward automating the process of creating well-behaved tensor datasets.
- **Practical algorithm**: We propose a practical, efficient, and effective algorithm that is able to produce high-quality tensors from raw data without incurring the combinatorial cost of the optimal solution. Our proposed method follows a greedy approach, where at each step, we decide whether different “slices” of the tensor are aggregated based on a variety of intuitive functions that characterize the “goodness of structure” locally.
- **Experimental evaluation**: We extensively evaluate our proposed method on synthetic, semi-synthetic, and real data where we use popular heuristic measures of structure goodness to measure success. Furthermore, we conduct a data mining case study on a large real dataset of crime over time in Chicago, where we identify interpretable hidden patterns in multiple time resolutions.

We make our implementation publicly available^1^ in order to encourage reproducibility of our results.

## 2. Problem formulation

### 2.1. Tensor definition and notations

Tensors are multi-dimensional extensions of matrices, and tensor decompositions are a class of methods that extract latent structure from tensor datasets by extending techniques such as principal component analysis and singular value decomposition. The different “dimensions” of a tensor are usually referred to as “modes.” In this paper, we focus on the CANDECOMP/PARAFAC (henceforth referred to as CP for brevity) decomposition (Carroll and Chang, 1970; Harshman, 1970), which is the “rank decomposition” of a tensor, i.e., the decomposition of an arbitrary tensor into a sum of *R* rank-one tensors. Mathematically, for a three-mode tensor **X**, the CP decomposition is $\underline{\text{X}}\approx \overset{R}{\underset{r=1}{\sum^{\text{​}}}}A(:,r)\circ B(:,r)\circ C(:,r)$, where ° is the generalized outer product. Matrices **A, B**, and **C** are called “factor matrices,” and each column corresponds to a latent pattern, directly relating an entity of the corresponding mode to a value that can be roughly construed as a soft clustering coefficient (Papalexakis et al., 2012). CP has arguably been the most popular tensor decomposition model in applications where the interest is to extract interpretable patterns for exploratory analysis, and thus, we adopt this decomposition model as our standard in this work. In the interest of space, we refer the reader to a number of available surveys (Kolda and Bader, 2009; Papalexakis et al., 2016; Sidiropoulos et al., 2017). We denote tensors as **X** and matrices as **X**, and we adopt Matlab-like notation for indexing.

### 2.2. Tensor decomposition quality

Unsupervised tensor decomposition, albeit very popular, poses a significant challenge: how can we state whether a computed decomposition is of “high quality,” and how can we go about defining “quality” in a meaningful way? Unfortunately, this happens to be a very hard problem to solve (Papalexakis, 2016) and defining a new measure of quality is beyond the scope of this paper. However, there has been a significant amount of work in that direction, which basically boils down to (1) model-based measures, where the quality is measured by how well a given decomposition represents the intrinsic hidden structure of the data and (2) extrinsic measures, where the quality is measured by how well the computed decomposition factors perform in a predictive task. However, extrinsic measures do not generalize, as they specialize to a particular labeled task, and in general, we cannot assume that labels will be available for the data at hand. Thus, in this study, we focus on model-based measures, which can provide a general solution.

In model-based measures, the most straightforward one is the fit, i.e., how well does the decomposition approximate the data under the chosen loss function, in a *low rank*. Low rank is key because the number of components (rank) has to be as small and compact as possible in order to lend itself to human evaluation and exploratory analysis. However, the fit is unstable and prone to errors especially in real and noisy data, thus the community has collectively turned its attention to more robust measures such as the Core Consistency Diagnostic (CORCONDIA for short) (Bro and Kiers, 2003), which measures how well the computed factors obey the CP model.

Both types of quality measure capture different elements of what an end-user would deem good in a set of decomposition factors. In this paper, we are going to use such popular measures of quality in order to characterize the quality of a given tensor dataset **X**. In order to do so, we assume that we have a function Q (**X**), which optimizes the quality measure *q*() for a given tensor over all possible decomposition ranks *R*^2^, i.e.,


(1) $$Q\left(\underline{\text{X}}\right)=\underset{R}{\max}q\left(\underline{\text{X}},A,B,C\right)$$

where **A, B**, and **C** are the *R*-column factor matrices for **X**. Finally, a useful operation is the *n*-mode product, where a matrix **W** is multiplied by the *n*-th mode of a tensor (predicated on matching dimensions in the *n*-th mode of the tensor and the rows of the matrix), denoted as **X**×_*n*_**W**. For instance, in an *I*×*J*×*K* tensor where *n* = 3 and **W** of size *K*×K^*^, the product **X**×_*n*_**W** multiplies all third mode slices of **X** with **W** and results in an *I*×*J*×K^*^ tensor.

### 2.3. The *Trapped Under Ice* problem

To give the reader an intuition of the problem, consider an example of a time-evolving graph that captures social activity over the span of some time. This example can be modeled as a three-mode tensor **X** of dimensions *I*×*J*×*K* where “sender” and “receiver” are the first two modes, “time” being the third mode, and non-zero entry in the tensor represents communications between users at a particular time. If the time granularity is extremely fine-grained (milliseconds or seconds), there might be only a handful of interactions/edges between nodes at a particular timestamp, resulting in an extremely sparse adjacency matrix for that timestamp, which, in turn, results in an extremely sparse tensor overall and to have a high tensor-rank as a result. In that case, **X** might not have any interpretable low-rank structure that can be exploited by CP. In this example, we assume that the third mode (time mode) is too fine-grained but in reality, any mode (one or more) can be extremely fine-grained. For example, in spatio-temporal data, where the first two modes are latitude and longitude and the third mode is time, all three modes can suffer from the same problem.

Given tensor **X** which is created using the “raw” granularities, how does one find a tensor (say **Y**) which has a better exploitable structure and hence can be decomposed into a meaningful latent structure. This is informally the *Trapped Under Ice* problem that we define here (which draws an analogy between the good structure that may exist within the data as being trapped under the ice and not visible by mere inspection). *Trapped Under Ice* has an inherent assumption that the mode in which we aggregate is ordered (e.g., representing time or space), thus permuting the third mode will lead to a different instance of the problem.

More formally we define our problem as follows:

Given a tensor **X** of dimensions *I*×*J*×*K* Find:

A tensor **Y** of dimensions *I*×*J*×K^*^ with K^*^ ≤ *K* such that

$$\underset{W}{\max}Q\left(\underline{\text{X}}\times_{3}W\right)$$

where Q is a measure of goodness and **W**(*i, j*) = 1 if slice *i* in tensor **X** is aggregated into slice *j* in the resulting tensor, otherwise **W**(*i, j*) = 0.

At first glance, *Trapped Under Ice* might look like a problem amenable to dynamic programming, since it exhibits the optimal substructure property. However, it lacks the overlapping subproblems property, which is across the set of different **W** matrices (e.g., two different matrices may have overlapping subproblems) but not within any single **W**. Thus, we still have to iterate over 2^*K*−1^ **W**'s, refer Section 2.4 for more details.

**Structure of** **W**: The matrix **W** has a special structure. For example, consider a three-mode tensor **X** of dimensions 10 × 10 × 10, with the third mode being the time mode. Suppose that the optimal level of aggregation for **Y** is K^*^ = 3.

In this case, **W** is of size 3 × 10 and an example of such a matrix is


$$\text{W}=\left[\begin{array}{cccccccccc} 1 & 1 & 1 & 0 & 0 & 0 & 0 & 0 & 0 & 0\\ 0 & 0 & 0 & 1 & 1 & 1 & 0 & 0 & 0 & 0\\ 0 & 0 & 0 & 0 & 0 & 0 & 1 & 1 & 1 & 1 \end{array}\right]$$

This **W** aggregates the first three slices of **X** to form the first slice of **Y**, then next three to form the second slice, and last four to form the third slice. No two **W** matrices will produce the same aggregation. They can have the same K^*^ but the order of aggregation of slices will be different.

### 2.4. Solving *Trapped Under Ice* optimally is hard

Solving *Trapped Under Ice* optimally poses a number of hurdles. First and foremost, the hardness of the problem depends on the definition of the function Q, and most reasonable and intuitive definitions are very hard to optimize since they are non-differentiable, non-continuous, and not concave. So far, in the literature, to the best of our knowledge, there are only heuristics for this quality function. Even so, those heuristic functions can only be evaluated on a single already fully-aggregated tensor, not a partially aggregated version thereof. Thus, *Trapped Under Ice* can only be solved optimally *via* enumerating all admissible solutions and choosing the best. In order to conduct this enumeration, we need to calculate the cardinality of the set of all **W** for a given instance of the problem.

**Lemma 1**. For an instance of a problem with *K* initial slices, the cardinality of the set of all **W** is 2^*K*−1^

Proof. To get K^*^ aggregated slices, there are K-1K*-1 ways to choose each of them leading to a different **W**. There are a number of ways that *K*−1 partition slots can be filled, partitioned by K^*^−1 blocks. In order to get the final number, we need to sum up over all potential K^*^:


$$\sum_{K^{*}=0}^{K-1}\left(\begin{array}{c} K-1\\ K^{*} \end{array}\right)=2^{K-1}$$

      □

The direct corollary of the above lemma is that solving optimally *Trapped Under Ice* requires calling the function Q *O*(2^*K*^) times, which is computationally intractable. There may be a small room for improvement by exploiting special structure in the set of all **W**, however, given discontinuities in our objective function Q, this is not a feasible alternative either. In this paper, we define proxy quality functions Q that lend themselves to partial evaluation on a partially aggregated solution, thus allowing for efficient algorithms Thus, in the next section, we propose a greedy approach that locally optimizes different criteria quality.

## 3. Proposed methods

In this section, we propose our efficient and effective greedy algorithm called ICEBREAKER which takes a tensor **X** as an input, which has been created directly from raw data, and has no exploitable structure and returns a tensor **Y**, which maximizes the interpretable and exploitable structure. The basic idea behind ICEBREAKER is to make a linear pass on the mode for which the granularity is suboptimal and using a number of intuitive and locally optimal criteria for the goodness of structure (henceforth referred to as *utility functions*), we greedily decide whether a particular slice across that mode needs to be aggregated^3^ into an existing slice or contains good-enough structure to stand on its own. ICEBREAKER can choose from a number of intuitive utility functions which are based on different definitions of good quality in matrices.

### 3.1. The ICEBREAKER algorithm

Algorithm 1 gives a high-level overview of ICEBREAKER. More specifically, the algorithm takes a three-mode tensor **X** of dimension *I*×*J*×*K* as an input and loops over all the *K* slices of tensor **X**. Two slices next to each other get aggregated into a single slice if a certain utility function *has stabilized*, i.e., if aggregating the two slices does not offer any additional utility (larger than a particular threshold), then the second slice should not be aggregated with the first and should mark the beginning of a new slice.

**Algorithm 1:** ICEBREAKER

**Input**: Tensor **X** of dimension *I*×*J*×*K*
**Output**: Tensor **Y** of dimension *I*×*J*×*K*_1_and matrix **W** of size *K*_1_×*K*
1: *i* = 1;*j* = 2
2: *previousValue* = *UtilityFunction*(*X*(:, :, *i*))
3: **while** *j* ≤ *K* **do**
4: *currentValue* = *UtilityFunction*(*sum*(*X*(:, :, *i*:*j*), 3)
5: **if** *previousValue*⋛*currentValue* **then**
6: j = j+1 {Aggregate Slice}
7: **else**
8: {Create a New Slice} Add a row in **W** with value as 1 for indices *i* to *j*−1. {Update indices for next candidate slice}
9: *i* = *j*; *j* = *j*+1;
10: previousValue = UtilityFunction(X(:,:,i));
11: **end if**
12: **end while**
13: **Y** = **X**×_3_**W**
14: return **Y** and **W** 2

Consider a three-mode tensor **X** with time as the third mode of dimension *I*×*J*×*K* is run through ICEBREAKER with a particular utility function. Our algorithm iterates over the time mode (*K* slices) and aggregates slices as decided by the utility function. ICEBREAKER is agnostic to the utility function used. Let us consider a slice that has been aggregated into a single slice from indices *i* to *j*−1 called the previous slice and another aggregated slice from indices *i* to *j* called a candidate slice. Both previous and candidate slices are passed to the utility function separately to obtain a value each called previous and current values, respectively. These values are compared (line 5 in Algorithm 1) to decide whether *j*^*th*^ slice is absorbed(line 6 in Algorithm 1) into previous slice or previous slice has stabilized and entry is added in *W* to indicate which indices of tensor **X** are aggregated together(line 8 − 9 in Algorithm 1). Now *jth* slice becomes the previous slice and aggregated slice of *j* and *j*+1 become the candidate slice, the whole process is repeated until all the slices are exhausted.

Note that ICEBREAKER's complexity is *linear* in terms of the slices *K* of the original tensor, and its overall complexity depends on the specific utility function used (which is called *O*(*K*) times).

#### 3.1.1. Utility functions

In this subsection, we summarize a number of intuitive utility functions that we are using in this paper. This list is by no means exhaustive and can be augmented by different functions (or function combinations) that capture different elements of what is good structure and can be informed by domain-specific insights.

1. **Norm:** We use multiple norm types to find the adaptive granularity of a tensor. For a given threshold, if the rate of change of norm between the previous and candidate slice is less than the threshold, the candidate slice is not selected. Our assumption, in this case, is that no significant amount of information is being added to the previous slice and is considered to have been stabilized. Matrix **W** is updated accordingly with indices of the previous slice (aggregated slices in the previous slice). Otherwise, the candidate slice is selected and the process continues until all the slices are exhausted. Different norms demonstrated in this work are Frobenius, 2-norm, and Infinity norm.
2. **Matrix rank:** In the case of matrix rank, we focus on the 95% reconstruction rank, which is typically much lower than the full rank of the data, but captures the essence of the number of components within the slice. In this case, we consider the previous slice to be stabilized if the matrix-rank of the previous slice decreases by the addition of a new slice, no more slices are added and an entry in matrix **W** is added. We keep aggregating slices if the matrix-rank of the slice is increasing or remains constant.
3. **Missing value prediction:** If a piece of data has a good structure, when we hide a small random subset of the data, the remaining data can successfully reconstruct the hidden values, under a particular model that we have chosen. To this end, we employ a variant of matrix factorization-based collaborative filtering (Koren, 2009) as a utility function to see how good is the aggregated matrix in predicting a certain percent of missing values. This utility function takes the percent of missing values as a parameter and hides those percent of non zeros values in the matrix. Our implementation of matrix factorization with Stochastic Gradient Descent tries to minimize the loss function: $\min_{U,V}\sum_{i,j\in \Omega }ℛℳSℰ\left(A_{ij}-U_{i,:}\cdot V_{:,j}\right)$ where **A** is a given slice, **U** and **V** are factor matrices for a given rank (typically chosen using the same criterion as the matrix rank above), and Ω is the set of *observed* (i.e., non-missing) values. In order to create a balanced problem, since we are dealing with very sparse slices, we conduct *negative sampling* where we randomly sample as many zero entries as there are non-zeros in the slice, and this ends up being the Ω set of observed values.

### 3.2. The ICEBREAKER++ algorithm

ICEBREAKER algorithm returns a tensor **Y** as an output that is considered to have an exploitable and better structure than the input tensor **X**. The idea behind ICEBREAKER++ is to recursively feed the output back to ICEBREAKER until the third mode is reduced to a single slice (matrix) or the dimension of the third mode does not change. ICEBREAKER algorithm returns a tensor associated with each utility function. Hence, if we used five utility functions, we would get 5 tensors associated with each of them. Now we select the tensor with the highest CP Fit (see Section 4.1), use that as input for ICEBREAKER, and we repeat this process until the stopping condition is met. The output of each iteration is a candidate tensor. In the end, we have multiple tensors (one for each iteration) which have different temporal resolutions, which can help us get a tensor with the optimal resolution based on the evaluation measures used. Algorithm 2 describes the process discussed in this section.

**Algorithm 2:** ICEBREAKER++.

**Input**: Tensor **Y** of dimension *I*×*J*×*K*
**Output**: One Tensor for each iteration
1: while *K* ≤ 1 do
2: for all Uitlity Functions do
3: [**Z**, **W**] = ICEBREAKER (**Y**)
4: end for
5: Select **Z** with the best Realtive fit {Third mode dimension}
6: *K*_1_ = *size*(**X**, 3)
7: if *K*_1_ = =*K* then
8: break;
9: else
10: *K* = *K*1
11: **Y** = **Z**
12: end if
13: end while
14: return one Tensor for each iteration 2

## 4. Experimental evaluation

In this section, we present a thorough evaluation of ICEBREAKER++ using variety of data, including synthetic, semi-synthetic, and real data. We empirically evaluate our analysis using a number of criteria described in detail below. We implement our method in Matlab using the tensor toolbox library (Bader et al., 2015).

### 4.1. Evaluation measures

When formulating the problem, we neither specify a quality function Q to be maximized nor did we use such a function in our proposed method. The reason for that is that we reserve the use of different quality functions as a form of evaluation. In particular, we use the two following notions of quality:

- **CP Fit:** To evaluate the effectiveness of our method, we compute the CP fit of the computed tensor for a particular rank with respect to the input tensor.

(2) $$Relative\;Fit=1-(\frac{||{\underline{\text{X}}}_{Input}-{\underline{\text{X}}}_{computed}|{|}_{F}}{||{\underline{\text{X}}}_{Input}|{|}_{F}})$$

- **CORCONDIA**: We employ AutoTen (Papalexakis, 2016), which essentially searches for the maximal number of components which attains a high CORCONDIA (Bro and Kiers, 2003) score, within a user-defined search space. AutoTen returns that number of components (i.e., the low rank) and the corresponding CORCONDIA score, which we use as our quality metric.

We should note at this point that the two quality measures above are far from continuous and monotonic functions, thus we do not expect that our method progressing the quality will monotonically increase. Thus, we calculate the quality for the final solution of ICEBREAKER++, and we reserve investigating whether monotonic and well-behaved quality functions exist for future work.

In our experiments, we used five utility functions (see Section 3.1.1) namely Frobenius norm, 2-norm, Infinity norm, Matrix Rank, and Missing Value Prediction. In the case of synthetic datasets, we ran all the utility functions once except for Missing Value Prediction which we ran 10 times. In case of both semi-synthetic and real datasets, in the interest of computational efficiency, we ran all the utility functions once.

### 4.2. Baseline methods

A naive way to find tensor **Y** can be by aggregating time mode based on some fixed intervals. If time granularity was in milliseconds, then combining one thousand slices to form slices of seconds granularity reduces the third dimension of tensor **X** from *K* to *K*/1, 000. This can be applied incrementally from seconds to minutes and so on to find a tensor that has some exploitable structure. We compare the resulting tensor **Y** determined by ICEBREAKER against tensors constructed with fixed aggregations. For fixed aggregation, we aggregate the temporal with a window size of 10, 100, and 1, 000 for synthetic data. For semi-synthetic and real datasets, we use appropriate time windows accordingly.

### 4.3. Performance for synthetic data

#### 4.3.1. Creating synthetic data

In order to create a synthetic dataset, we follow a two-step process:

1. We create a random sparse tensor of specific sparsity.
2. Subsequently, we randomly distribute (drawn from a uniform distribution) non zero entries in each slice over some fixed number of slice as explained in below example.

**Figure 2:**
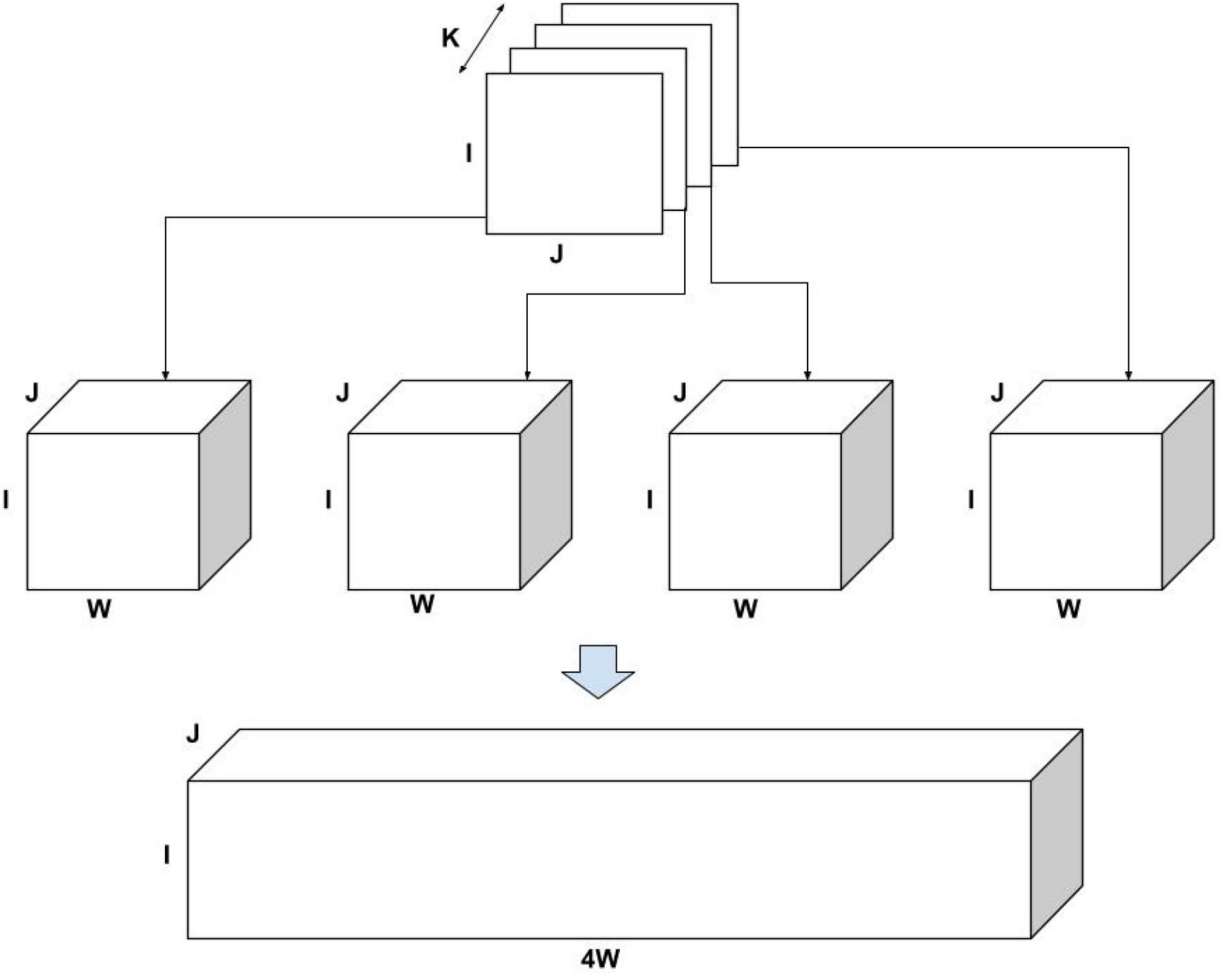
Creating synthetic data.

**Example:** Consider a three-mode tensor **X** of dimension *I*×*J*×*K*, for purpose of this example, consider *K* = 4 as shown in Figure 2. Now for each slice of size *I*×*J*, distribute randomly (drawn from Uniform distribution) all the non-zeros entries across *W* slices preserving the *I* and *J* indices, creating a tensor of the size *I*×*J*×*W*. Now append all the tensors in the same order as they appeared in the original tensor, we get a resulting tensor of size *I*×*J*×4*W*, which is used as an input for ICEBREAKER. Thus, if the original tensor is of size *I*×*J*×*K* and bucket size *W*, the resulting tensor is of the size *I*×*J*×*KW* approximately^4^. Table 1 shows the synthetic data used for experiments.

**Table 1:** Table of synthetic datasets analyzed.

**Dataset**	**Original dimension**	**Window size (*W*)**	**Approximate final dimension**	**Number of datasets**
SD1	100 × 100 × 10	50	100 × 100 × 500	10
SD2	100 × 100 × 100	50	100 × 100 × 5, 000	10

#### 4.3.2. Results for synthetic data

**Figure 3:**
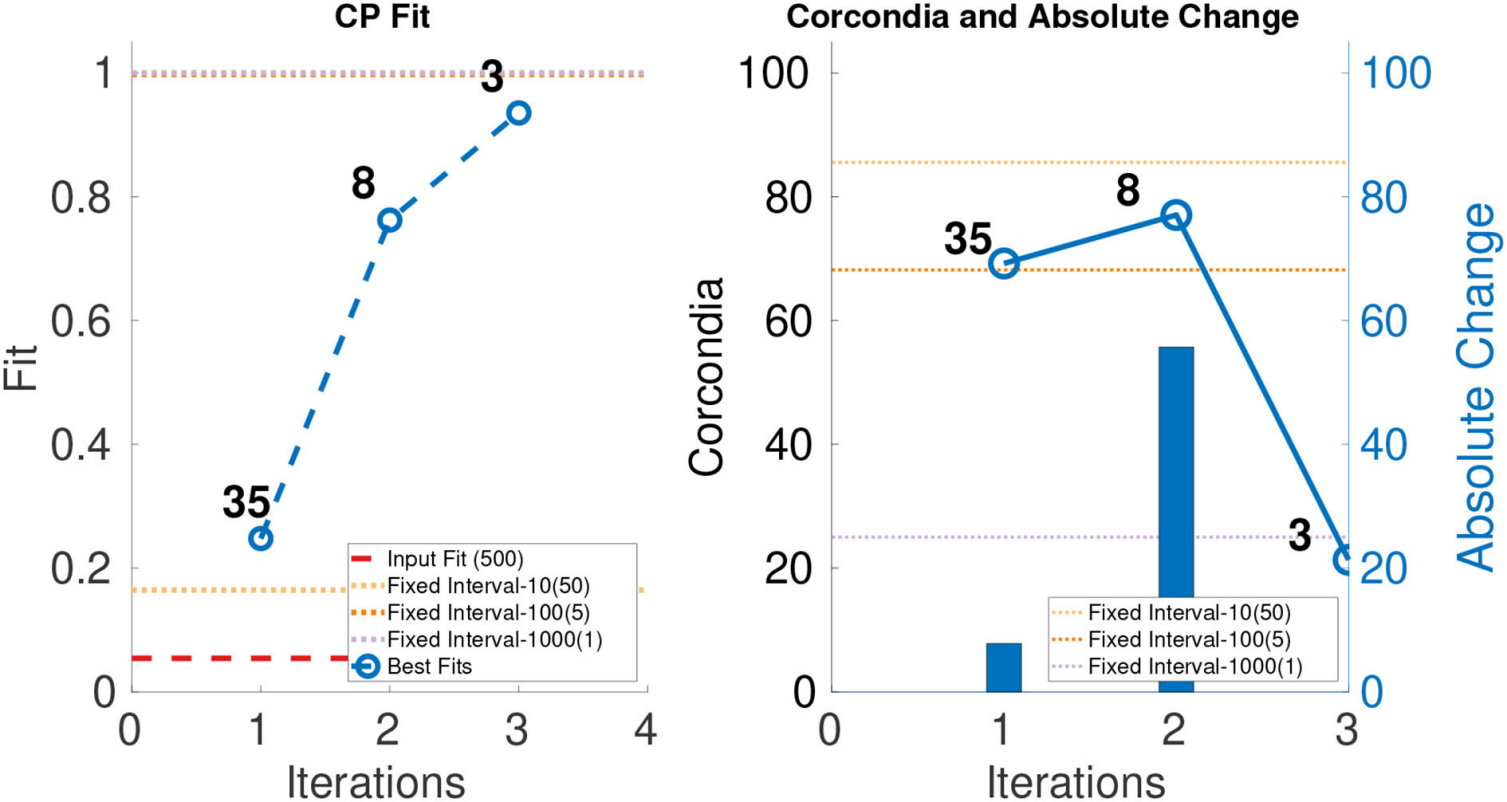
CANDECOMP/PARAFAC (CP) fit and Core Consistency Diagnostic (CORCONDIA) of best fit tensor and its absolute change at each iteration for SD1.

**Figure 4:**
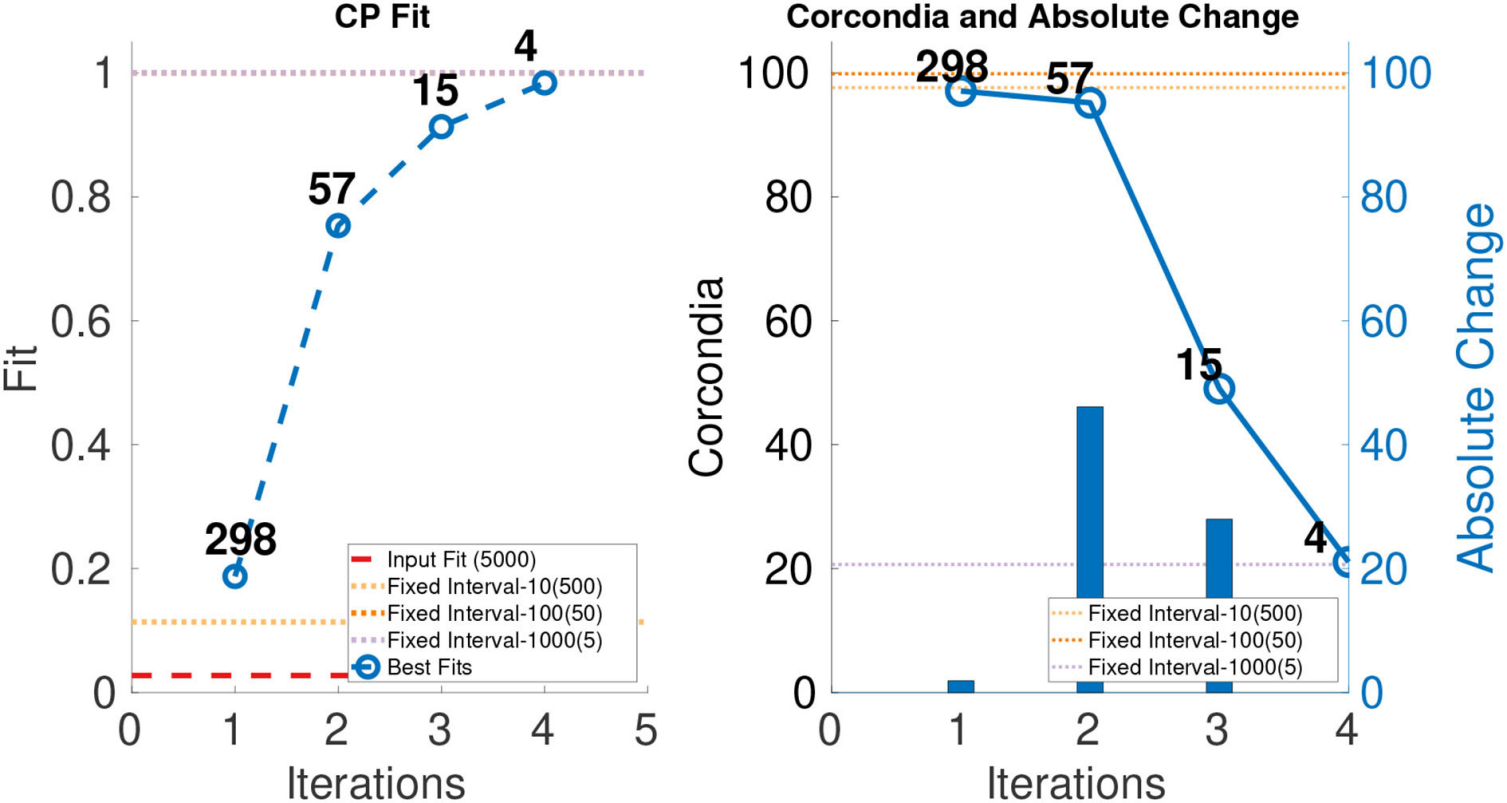
CP fit and CORCONDIA of best fit tensor and its absolute change at each iteration for SD2.

In order to evaluate the performance of ICEBREAKER++, we measure CORCONDIA and fit it on 10 synthetic datasets for both types of datasets as mentioned in Table 1. In interest of conserving space, we only show one set of results for both synthetic datasets. The leftmost part of Figures 3, 4 show the best fit at end of each iteration. The number on top of the dots represents the dimension of the third mode after each iteration. The dotted line in the plot shows the fit of the input tensor and fixed intervals tensor^5^. The rightmost part of Figures 3, 4 show the CORCONDIA computed at the end of each iteration and the absolute change of CORCONDIA. Absolute change of CORCONDIA is computed as shown below:


$$\begin{array}{l} abs\left(corcondia\left(j+1\right)-corcondia\left(j\right)\right) \end{array}$$

The dotted line in the plot represents CORCONDIA value for the fixed intervals tensor. When there is a sudden drop in the value of CORCONDIA, we consider the iteration before as a suitable candidate for tensor analysis. In the case of SD1 that would be iteration number 2 and the resulting tensor of size 100 × 100 × 8. In the case of SD2 that would also be iteration number 2 and the resulting tensor of size 100 × 100 × 57.

### 4.4. Performance for semi-synthetic data

#### 4.4.1. Creating semi-synthetic data

In this study, we used the Enron dataset (Priebe et al., 2006; Bader et al., 2007), which is a dataset of the number of email exchanges between employees spread over 44 months. Each month is represented by a matrix. To create the semi-synthetic data, we use step 2 as described in the generation of synthetic case. We take the non-zero elements and randomly distribute non zero entries in each slice over some fixed number of the slice. For this dataset, we converted the monthly data into weekly, daily, and hourly data. Non-zero entries in each slice were distributed over four different candidate slices for creating the weekly dataset (roughly approximating 4 weeks as a month). In the case of daily, each slice of monthly data was distributed over 30 different slices as mentioned in Table 2 and finally in the case of hourly, each non zero entry in the monthly slice was distributed over 720 slices (24 × 30).

**Table 2:** Table of semi-synthetic datasets analyzed.

**Dataset**	**Original dimension**	**Window size (*W*)**	**Approximate final dimension**
Enron weekly	184 × 184 × 44	4	184 × 184 × 176
Enron daily	184 × 184 × 44	30	184 × 184 × 1, 320
Enron hourly	184 × 184 × 44	720	184 × 184 × 31, 680

#### 4.4.2. Results for semi-synthetic data

**Figure 5:**
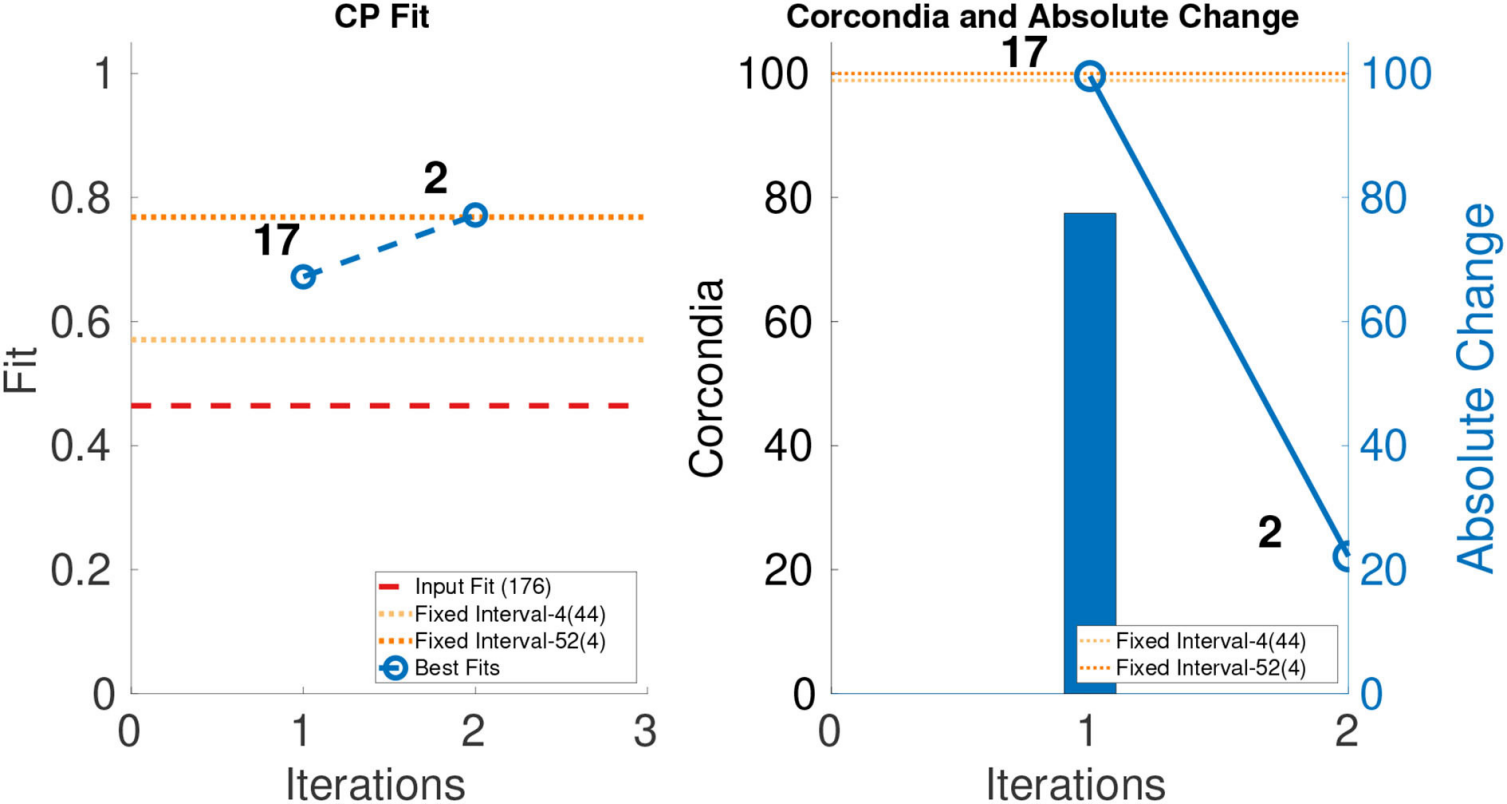
CP fit and CORCONDIA of best fit tensor and its absolute change at each iteration for Enron Weekly.

**Figure 6:**
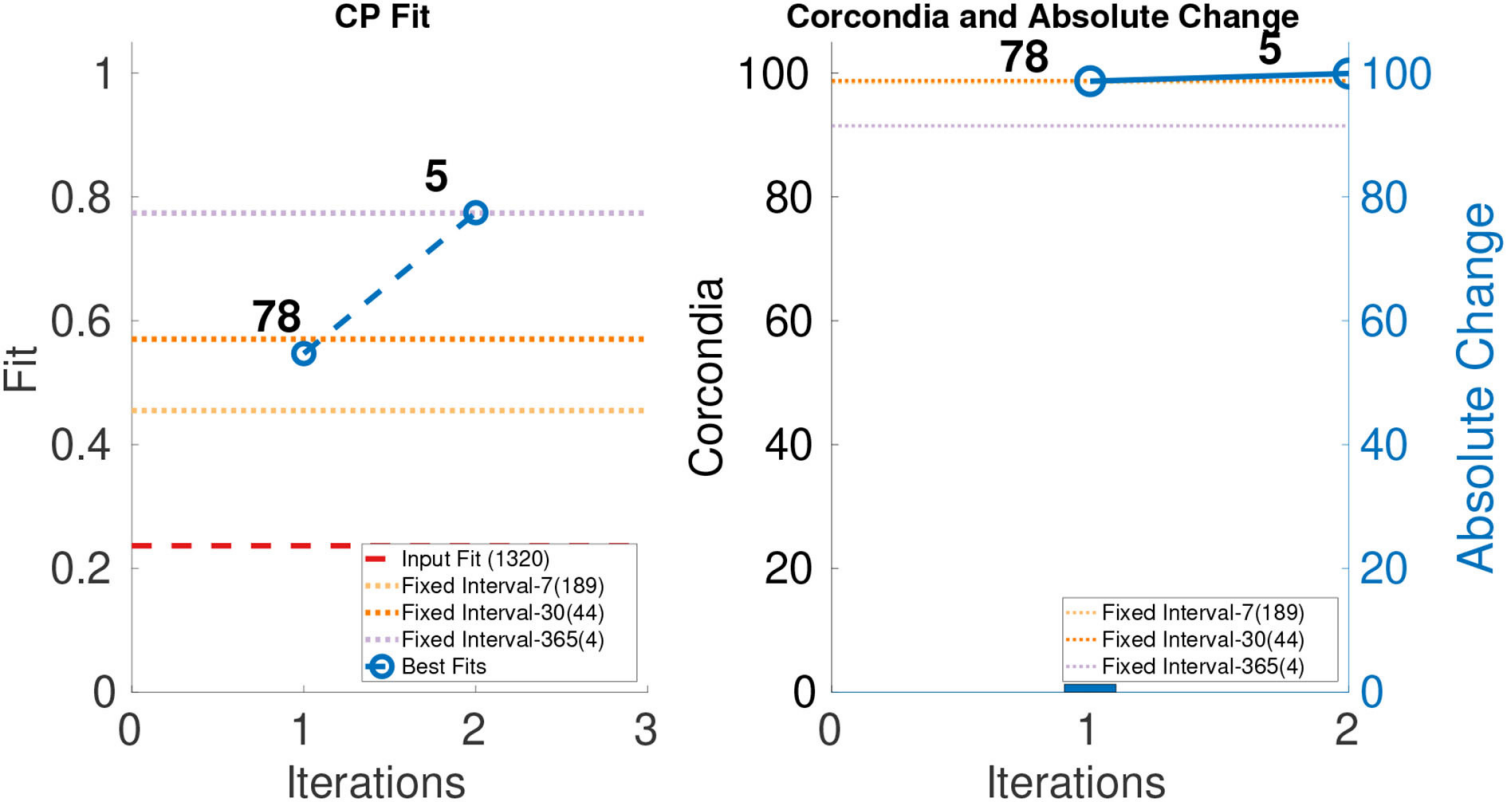
CP fit and CORCONDIA of best fit tensor and its absolute change at each iteration for Enron Daily.

**Figure 7:**
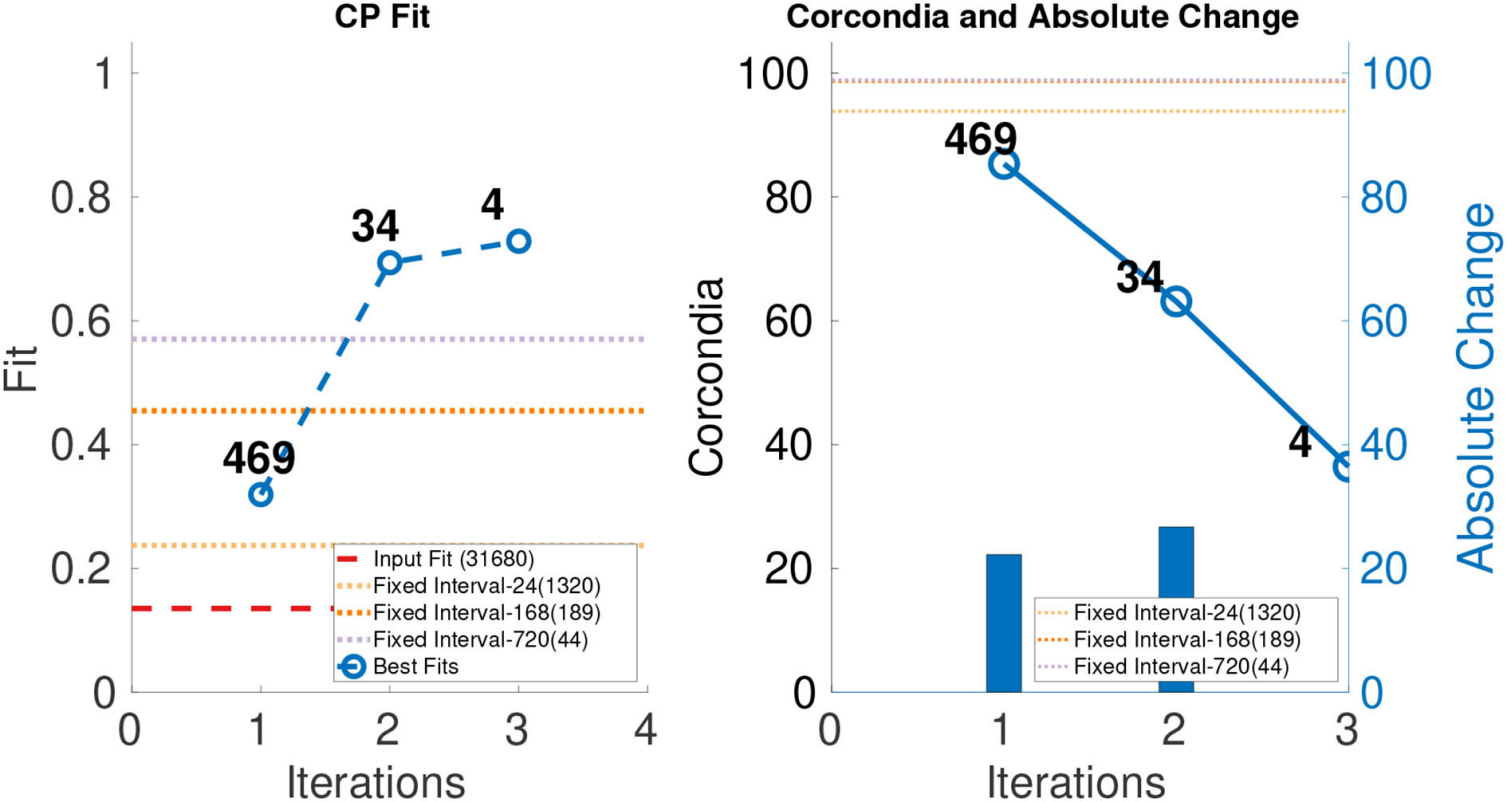
CP fit and CORCONDIA of best fit tensor and its absolute change at each iteration for Enron Hourly.

The leftmost parts of Figures 5–7 show the fit of different iterations and the rightmost part of the Figures 5–7 show the CORCONDIA computed at different iterations. In the case of Enron Weekly, we see a sudden drop in CORCONDIA after iteration 1 as shown in Figure 5 and the corresponding tensor is of size 184 × 184 × 17. In the case of Enron Daily, we do not see a significant change in CORCONDIA values in two iterations and corresponding tensors are of size 184×184×78 and 184×184×5 giving us tensors of different granularity.

In the case of Enron Hourly, we see a drop in CORCONDIA after iterations 1 and 2 as shown in Figure 7. In this case, practitioner can make choice between a tensor of resolution 184×184×469 or 184×184×34 depending on what evaluation metric they value more, fit, CORCONDIA, or both. Tensor after iteration 2 (184×184 ×34) seems to have a good score for both fit and CORCONDIA whereas the Tensor after iteration 1 has a good CORCONDIA score but not a good CP fit.

### 4.5. Data mining case study

#### 4.5.1. Chicago crime dataset

For our case study, we use a dataset provided by the city of Chicago^6^ that records different types of crime committed in different areas of the city over a period of time (Smith et al., 2017). The tensor we create has modes (area, crime, and timestamp), where “community area” and “crime” are discretized by the city of Chicago, and “timestamp” is the coarsely aggregated (hourly) timestamp. The dates that we focused was on a span of 7 years, from 13 December 2010 to 11 December 2017.

**Figure 8:**
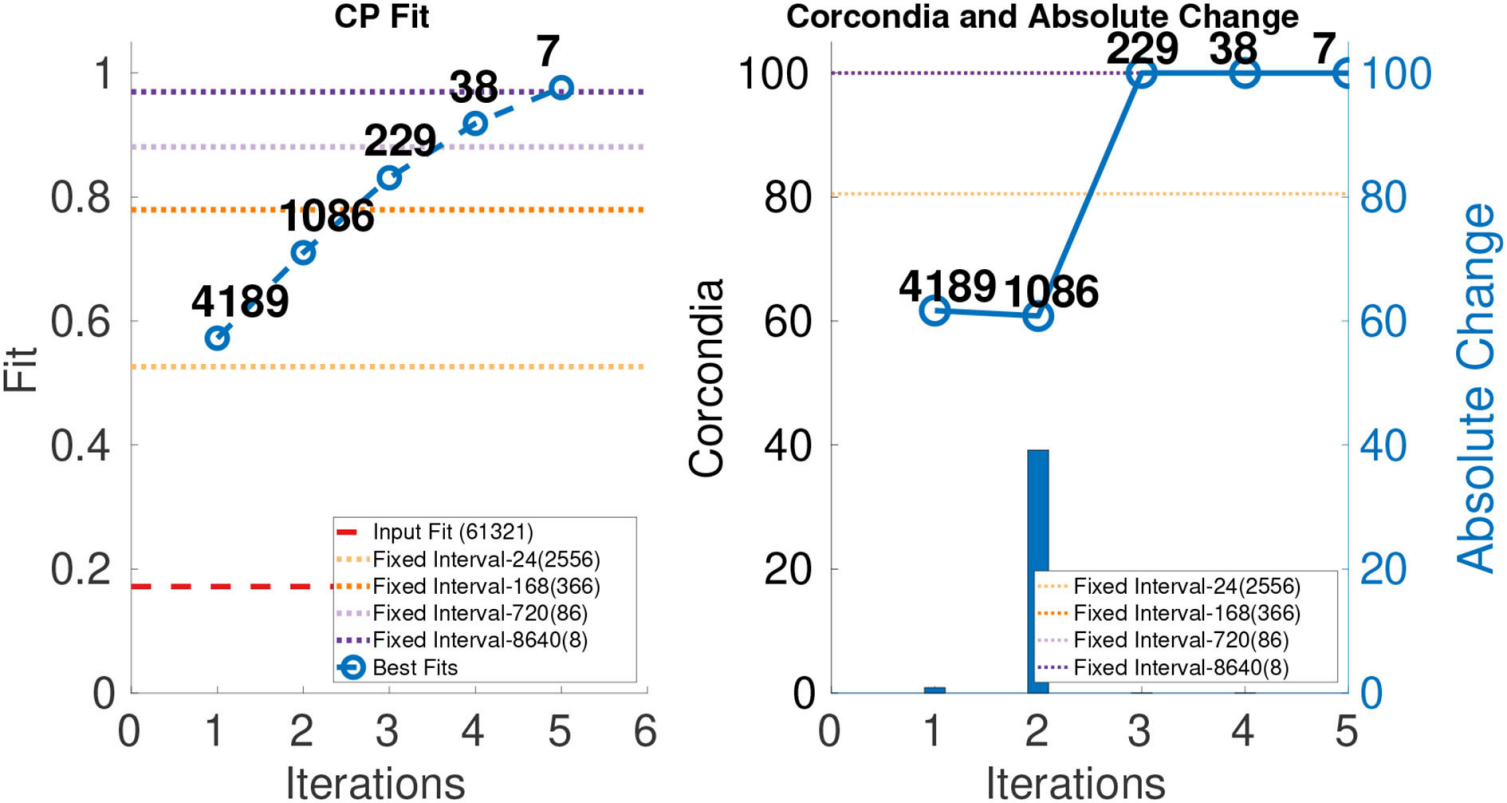
CP fit and CORCONDIA of best fit tensor and its absolute change at each iteration for Chicago Crime Dataset.

We ran ICEBREAKER++ on this dataset which is of size 77 × 32 × 61, 321, and in the right most part of Figure 8, we show its CORCONDIA for each iteration and we observe that iterations 3, 4, and 5 have high values of CORCONDIA, which would suggest they offer a resolution with an exploitable structure. Iterations 1 and 2 also have decent CORCONDIA values. Given these two ranges of CORCONDIA values, we decided to drill down and look into the actual tensor components that can be extracted from those different tensors. In the interest of space, we took the tensor returned by iteration 2 as **X**_1_, the tensor **X**_2_ and tensor **X**_3_ are returned by iterations 3 and 4, respectively. Tensor **X**_1_ contains three high-quality components, whereas **X**_2_ and **X**_3_ contain two.

**Figure 9:**
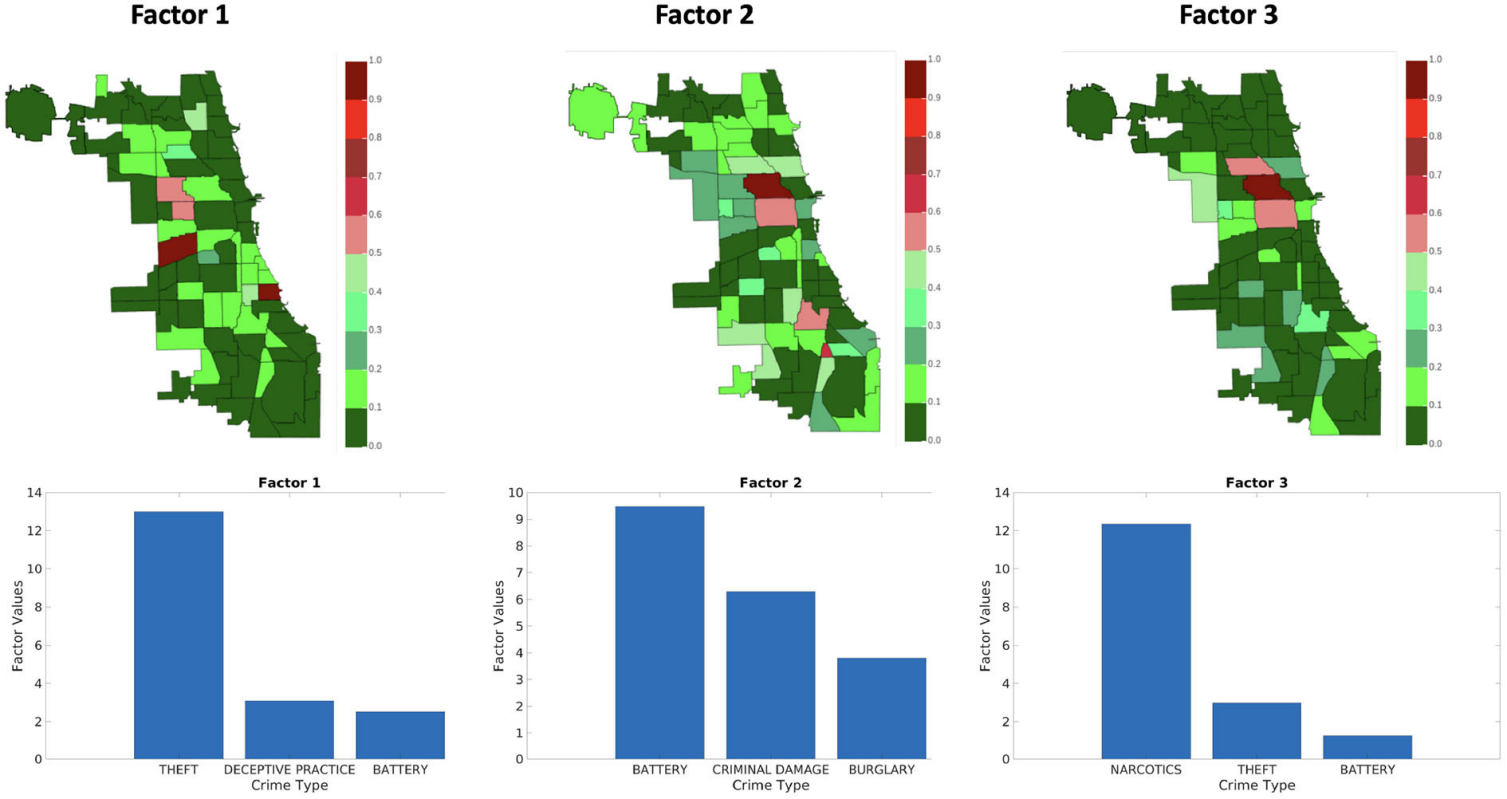
Analyzing the Chicago data from iteration-2 (**X**_1_). Chicago heatmap value ranges from 0.0 to 1.0.

**Figure 10:**
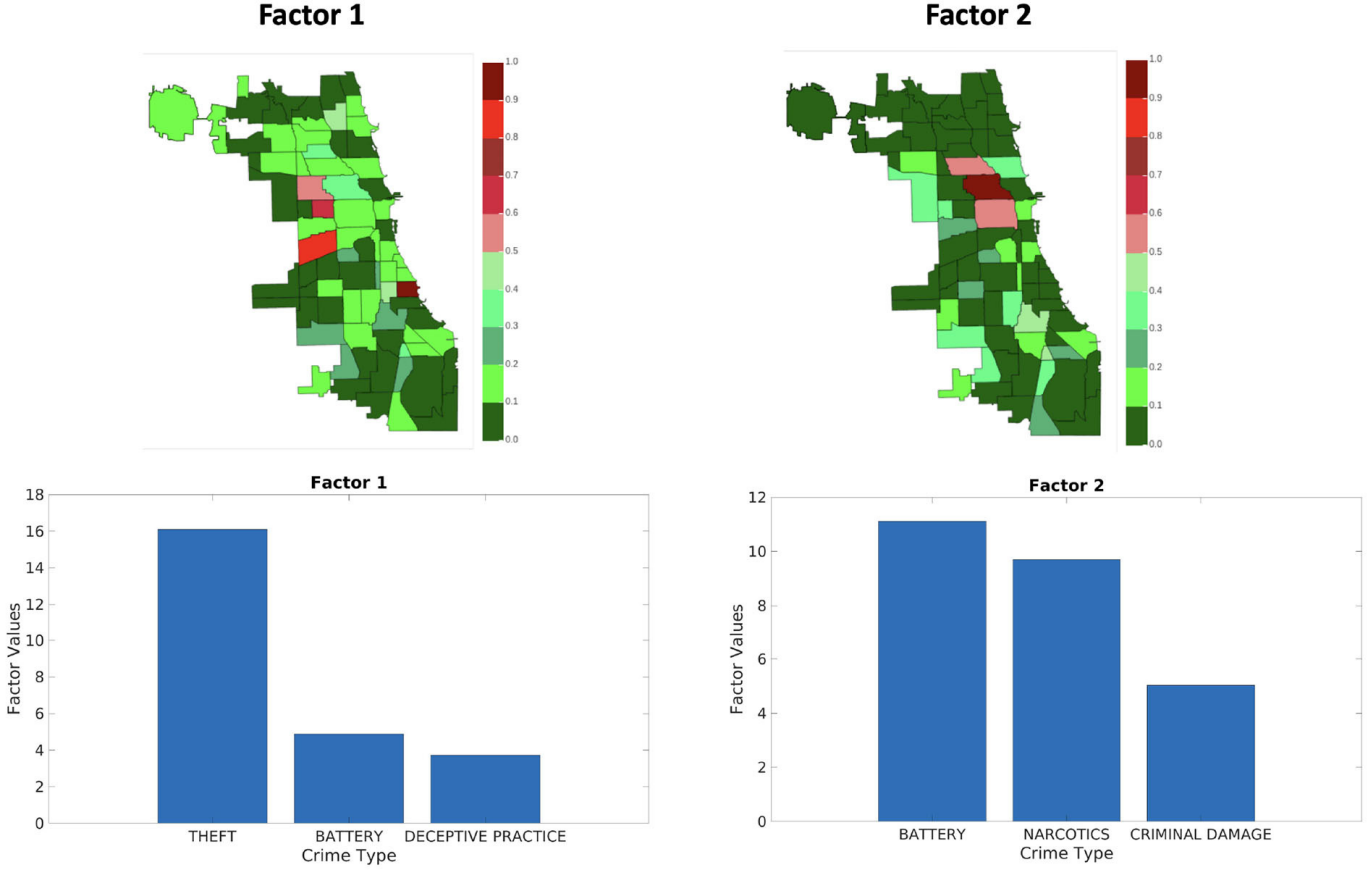
Analyzing the Chicago data from iteration-3 (**X**_2_). Chicago heatmap value ranges from 0.0 to 1.0.

**Figure 11:**
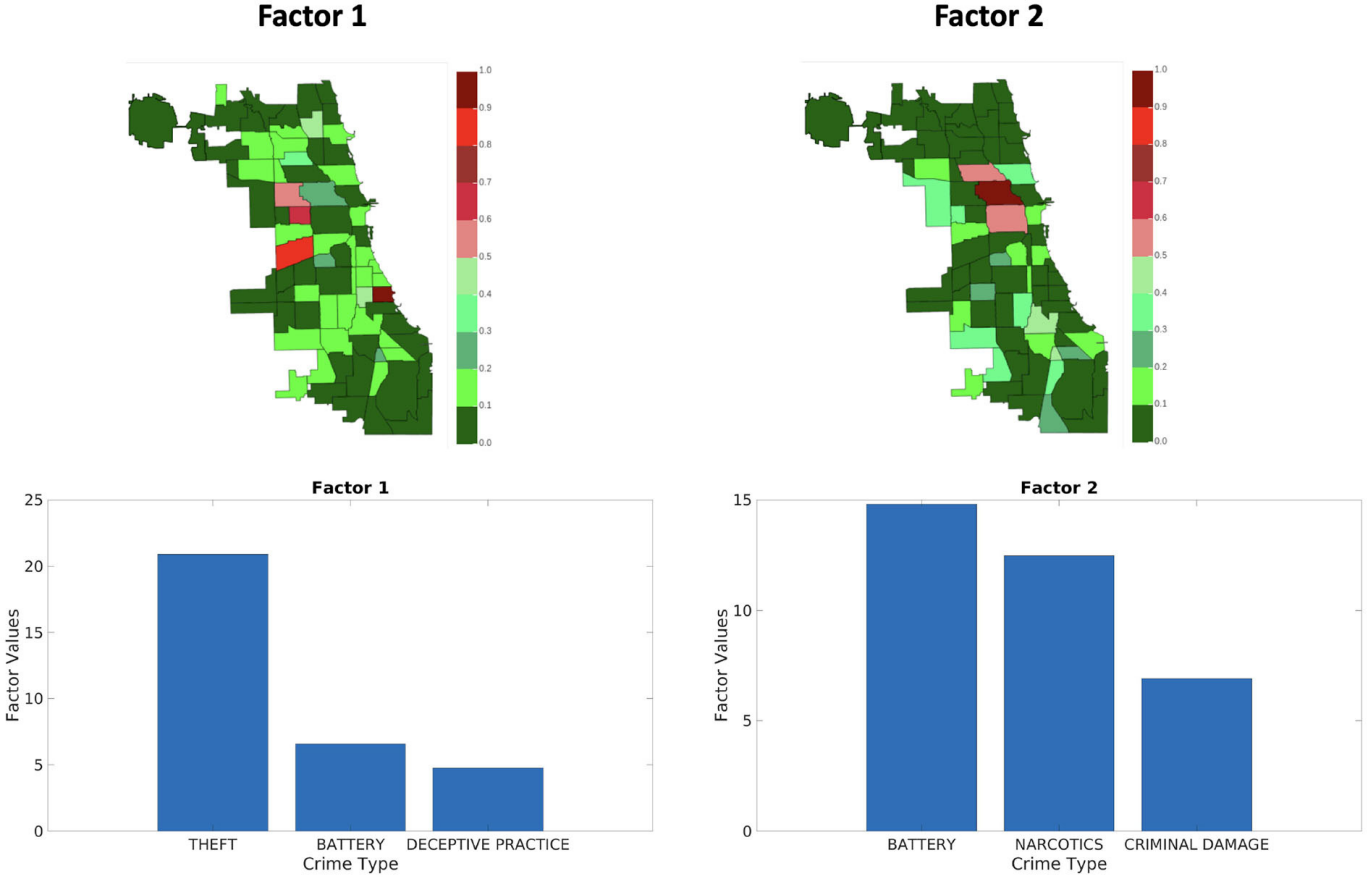
Analyzing the Chicago data from iteration-4 (**X**_3_). Chicago heatmap value ranges from 0.0 to 1.0.

Figures 9–11 shows sets of patterns^7^ for **X**_1_, **X**_2_, and **X**_3_, respectively: interestingly, factor 1 of **X**_1_ and factor 1 of **X**_2_ pertain to similar spatial and criminal pattern. As shown in Figures 10, 11, we observed that both factors of tensors **X**_2_ and **X**_3_ pertain to similar spatial and criminal patterns. In summary, tensors **X**_1_, **X**_2_, and **X**_3_ capture similar interpretable patterns over different temporal resolutions.

#### 4.5.2. Comparison against fixed aggregation

A natural question is whether the results are qualitatively “better” than the ones by a fixed aggregation. Answering this question heavily depends on the application at hand, however, here we attempt to quantify this in the following way: intuitively, a good set of components offers more *diversity* in much of the data it covers. For instance, a practitioner would prefer a set of results for the Chicago crime dataset where the components span most of the regions of the city and uncover diverse patterns of crime, over a set of components that seem to uncover a particular type of crime. Even though there may be a number of confounding factors, aggregating on a regular time interval may be very good in capturing periodic activity (in this example, crime that exhibits normal periodicity happens to coincide with the aggregation resolution we have chosen), whereas aggregating adaptively may help discover the structure that is more erratic and more surprising. In order to capture this and test this hypothesis, we compute the coverage of entities for the first and second mode of the tensor (i.e., areas of Chicago and crime types in this example) in all the discovered components: for each component, we measure the top-k entities, and through that, we compute the empirical probability distribution of all entities in the results. A more preferable set of results will have higher coverage, resulting in distribution with higher entropy. In Table 3, we show the entropy for both modes 1 and 2 forICEBREAKER++ and for the different fixed aggregations (averaged over 10 different runs), where ICEBREAKER++ overall offers more diverse patterns in both space and criminal activity.

**Table 3:** The entropy of top-3 components in factors for the area and crime type.

	**Iteration**	**Iteration**	**Iteration**	**Iteration**	**Iteration**	**Fixed**	**Fixed**	**Fixed**	**Fixed**
	**1**	**2**	**3**	**4**	**5**	**Interval-24**	**Interval-168**	**Interval-720**	**Interval-8640**
Area	**2.8554**	2.6810	2.5850	2.5850	2.5850	2.7255	2.5850	2.5850	2.5850
Crime	**2.8783**	2.7255	2.2516	2.2516	2.0850	2.4362	2.2516	2.2516	2.1183

Black color is best icebreaker entropy and red color is best fixed interval entropy.

## 5. Related work

To the best of our knowledge, this is the first attempt at formalizing and solving this problem, especially as it pertains to the tensor and multi-aspect data mining domain. Nevertheless, there has been significant amount of work on temporal aggregations in graphs (Sun et al., 2007; Sulo et al., 2010; Soundarajan et al., 2016) and in finding communities in temporal graphs (Gorovits et al., 2018). In the graph literature, the closest work to ours is Soundarajan et al. (2016), in which the authors look at aggregating stream of temporal edges to produce a sequence of structurally mature graphs based on a variety of network properties.

In the tensor literature, Almutairi et al. (2021) solved the inverse of this problem, where the goal is to disaggregate a tensor. Concurrently to our work, Kwon et al. (2021) developed a streaming CP decomposition that works on the original granularity of the data, instead of preprocessing the tensor in order to identify one or more optimal aggregations. We reserve a full investigation of connections between our problem formulation and Kwon et al. (2021)'s study for future work.

## 6. Conclusions

In this study, we are, to the best of our knowledge, the first to define and formalize the *Trapped Under Ice* problem in constructing a tensor from raw sparse data. We demonstrate that an optimal solution is intractable and subsequently proposed ICEBREAKER++, a practical solution that is able to identify good tensor structure from raw data and construct tensors from the same dataset that pertain to multiple resolutions. Our experiments demonstrate the merit of ICEBREAKER++ in discovering useful and high-quality structures and providing tools to data analysts in automatically extracting multi-resolution patterns from raw multi-aspect data. In the future, we will work toward extending ICEBREAKER in cases where more than one mode is *Trapped Under Ice* (naively one can apply ICEBREAKER to each mode sequentially, but this disregards joint variation across modes) and extend ICEBREAKER for higher-order tensors.

## Data availability statement

All publicly available data and the original contributions presented the original contributions presented in the study are included in the article/supplementary material, further inquiries can be directed to the corresponding author/s.

## Author contributions

RP: design, implementation, experimental evaluation, and writing. EG: design and writing. EP: design, writing, and primary research advisor of RP and EG. All authors contributed to the article and approved the submitted version.

## Funding

This research was supported by the National Science Foundation under CAREER grant no. IIS 2046086 and the Department of the Navy, Naval Engineering Education Consortium under award no. N00174-17-1-0005. It was sponsored by the Combat Capabilities Development Command Army Research Laboratory and was accomplished under Cooperative Agreement Number W911NF-13-2-0045 (ARL Cyber Security CRA).

## Conflict of interest

The authors declare that the research was conducted in the absence of any commercial or financial relationships that could be construed as a potential conflict of interest.

## Publisher's note

All claims expressed in this article are solely those of the authors and do not necessarily represent those of their affiliated organizations, or those of the publisher, the editors and the reviewers. Any product that may be evaluated in this article, or claim that may be made by its manufacturer, is not guaranteed or endorsed by the publisher.

## Author disclaimer

The views and conclusions contained in this document are those of the authors and should not be interpreted as representing the official policies, either expressed or implied, of the Combat Capabilities Development Command Army Research Laboratory or the US Government. The US Government is authorized to reproduce and distribute reprints for government purposes not withstanding any copyright notation here on.

## Footnotes

^1^ https://github.com/ravdeep003/adaptive-granularity-tensors

^2^In practice, this is done over a small number of low ranks, since low-rank structure is desirable.

^3^For the purposes of our work, we use matrix addition as aggregation of slice but this might not be the case and would depend on the problem domain. Other aggregation functions that can be used are OR, min, max, depending on the application domain (e.g., binary data).

^4^The number of slice can be less than *KW*, since slice for each non-zero value is selected randomly, there can be a case where a slice is not selected.

^5^The number in the parenthesis represents the dimension of the third mode for that tensor.

^6^ https://data.cityofchicago.org/Public-Safety/Crimes-2001-to-Present/ijzp-q8t2

^7^We omit plotting the temporal mode since we lack external information that we can potentially correlate it with, however, an analyst with such side information can find the different time resolutions of **X**_1_, **X**_2_, and **X**_3_ useful.
